# Small heat shock proteins in neurodegenerative diseases

**DOI:** 10.1007/s12192-020-01101-4

**Published:** 2020-04-22

**Authors:** Leen Vendredy, Elias Adriaenssens, Vincent Timmerman

**Affiliations:** Department of Biomedical Sciences and Institute Born Bunge, Peripheral Neuropathy Research Group, University of Antwerp, Antwerp, Belgium

**Keywords:** Small heat shock proteins, Hereditary peripheral neuropathies, Diseases of the central nervous system, Neurodegeneration, Protein aggregation

## Abstract

Small heat shock proteins are ubiquitously expressed chaperones, yet mutations in some of them cause tissue-specific diseases. Here, we will discuss how small heat shock proteins give rise to neurodegenerative disorders themselves while we will also highlight how these proteins can fulfil protective functions in neurodegenerative disorders caused by protein aggregation. The first half of this paper will be focused on how mutations in HSPB1, HSPB3, and HSPB8 are linked to inherited peripheral neuropathies like Charcot-Marie-Tooth (CMT) disease and distal hereditary motor neuropathy (dHMN). The second part of the paper will discuss how small heat shock proteins are linked to neurodegenerative disorders like Alzheimer’s, Parkinson’s, and Huntington’s disease.

## Introduction

Small heat shock proteins (sHSPs) are molecular chaperones whose canonical function is to preserve cellular proteostasis. Unlike heat shock proteins with an ATPase domain (e.g., HSP70), sHSPs are ATP-independent chaperones that recognize and bind unfolded or misfolded proteins. Target proteins are kept in a folding-competent state until ATP-dependent heat shock proteins complete the refolding process (Haslbeck et al. 2005). When proper folding fails, sHSPs aid in the clearance of these target proteins by routing them to one of the degradation systems. The family comprises 10 members (HSPB1–10), which all share a characteristic, conserved α-crystallin domain (ACD) flanked by variable N-terminal and C-terminal domains (Table 1). The tissue distribution depends on the individual protein and varies from ubiquitous to tissue specific. A remarkable feature of most sHSPs, which has been considered important for their function, is their ability to form a wide range of oligomers (Haslbeck and Vierling 2015). These oligomers are assembled from dimeric building blocks that interact with each other and typically range from 12 to greater than 32 subunits. The chaperone activity of most sHSPs has been characterized in vitro (Mymrikov et al. 2017), but there is no consensus yet on the active sHSP species, as chaperone activity has been assigned to large oligomers (Franzmann et al. 2005; Bepperling et al. 2012), small oligomers (Stengel et al. 2010; Fleckenstein et al. 2015), dimers (Van Montfort et al. 2001), and even monomers (Almeida-Souza et al. 2010; Alderson et al. 2019).

**Table 1:** Members of the sHSP family and related diseases

Name	Expression	Associated disease(s)	Target disease aggregates
HSPB1 (HSP27)	Ubiquitous	CMT2, dHMN, cancer, ischemia and reperfusion	Aβ, tau, α-synuclein, huntingtin, SOD1
HSPB2 (MKBP)	Heart and skeletal muscle	/	Aβ^a^
HSPB3 (HSPL27)	Heart, brain, skeletal and smooth muscle	CMT2, dHMN, myopathy	/
HSPB4 (CRYAA)	Eye lens, skeletal muscle	Cataract	/
HSPB5 (CRYAB)	Ubiquitous	Cataract, (cardio)myopathy, ischemia and reperfusion	Aβ, tau, α-synuclein, Ataxin-3, huntingtin, SOD1
HSPB6 (HSP20)	Ubiquitous	Cardiomyopathy, ischemia and reperfusion	Aβ, huntingtin
HSPB7 (cvHSP)	Heart and skeletal muscle	N.A.	Ataxin-3, huntingtin
HSPB8 (HSP22)	Ubiquitous	CMT2, dHMN, myopathy	Tau, Ataxin-3, huntingtin, TDP-43, C9orf72, SOD1
HSPB9 (CT51)	Testis	/	Ataxin-3, huntingtin
HSPB10 (ODF1)	Testis	/	/

^a^This result stems from a double HSPB2/HSPB5 knock-out mouse model

Despite their multifunctionality and ubiquitous expression, mutations in HSPB1 and HSPB8 affect specifically the peripheral nervous system causing the axonal form of Charcot-Marie-Tooth neuropathy (CMT2) and/or distal hereditary motor neuropathy (dHMN) (Evgrafov et al. 2004; Irobi et al. 2004). Later, through a candidate gene approach, it was reported that also HSPB3 may cause dHMN (Kolb et al. 2010). dHMN are a group of clinically and genetically heterogeneous diseases characterized by degeneration of motor neurons in the peripheral nervous system. Patients experience progressive motor impairment, weakness, and atrophy of distal limb muscles, suggesting a length-dependent neurodegeneration affecting primarily the longest motor axons. Sensory neurons are usually not involved in dHMN. In contrast, CMT patients present with both motor and sensory symptoms. However, many CMT2 patients present with only minor sensory involvement, making it difficult to distinguish dHMN from CMT2 (Harding and Thomas 1980). Intriguingly, genetic overlap is observed between CMT and dHMN as both diseases can be caused by mutations in the same gene and even by the same mutation. Moreover, it was recently demonstrated that some patients with HSPB1 and HSPB8 mutations may have rare forms of ALS or distal myopathy, respectively, further supporting the genetic and clinical heterogeneity (Dierick et al. 2007; Capponi et al. 2016; Ghaoui et al. 2016; Echaniz-Laguna et al. 2017b). To date, no effective treatment is available to delay or cure patients with sHSP mutations. Understanding the molecular mechanisms of sHSPs is essential for the development of new therapies, which represents a big challenge due to the wide variety of functions exerted by these proteins.

While mutations in certain sHSPs give rise to degeneration of the peripheral nervous system, degeneration of the central nervous system may also involve sHSPs. Central nervous system disorders are often caused by mutations in genes other than sHSPs, which render the produced protein more susceptible to aggregation. These protein aggregates are typically one of the hallmarks of these diseases. As sHSPs may recognize and prevent these proteins from aggregating, sHSPs were found to be involved in many neurodegenerative disorders and became interesting targets for protective strategies (Kampinga and Bergink 2016). Typical examples of such protein aggregation diseases are Alzheimer’s, Parkinson’s, and Huntington’s disease.

In this paper, we will first summarize how mutations in HSPB1, HSPB3, and HSPB8 cause hereditary peripheral neuropathies. In addition, as part of their core function, sHSPs prevent and counter other proteins from aggregating. Manipulation of sHSP expression or activity has become an attractive approach to ameliorate neurodegenerative diseases, which we will outline in the second part of this paper.

## Mutations in sHSPs causing neurodegenerative diseases

### HSPB1

**Fig. 1:**
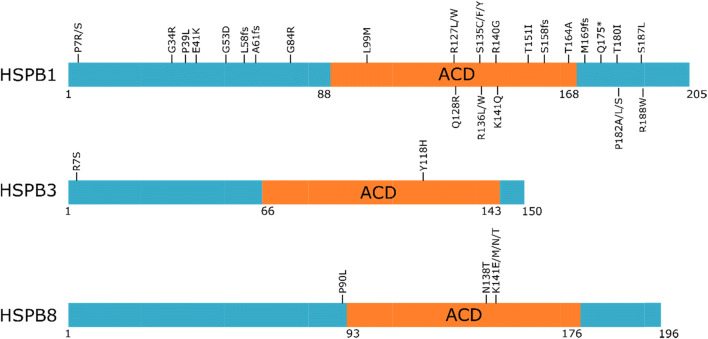
Overview of all mutations in HSPB1, HSPB3, and HSPB8 reported to cause CMT2/dHMN. *Premature stop codon. fs, frameshift

Patients with HSPB1 mutations usually present with progressive weakness of the legs, with bilateral foot drop often arising as the first symptom. The age of onset is usually in the second decade of life, although onsets up to the seventh decade of life have also been reported (Harding and Thomas 1980; Capponi et al. 2011; Echaniz-Laguna et al. 2017a; Rossor et al. 2017). Disease progression is slow and a significant percentage of patients develop upper limb weakness over the years, leading to loss of ambulation, resulting eventually in wheelchair dependence. Patients may also suffer from mild sensory involvement, although the degree of involvement is highly variable and can even differ between affected members of the same family (Rossor et al. 2017). Other common features include mildly elevated creatine kinase levels, foot deformities, and thigh and hand weakness; and CNS involvement was observed in 5–10% of the patients (Echaniz-Laguna et al. 2017a, Rossor et al. 2017). So far, over 30 different mutations have been found in the *HSPB1* gene leading to inherited peripheral neuropathies (Fig. 1, Table 2). Depending on the protein domain where the mutations are located, the impact on protein function and the clinical outcome of the patient can differ. The majority of the mutations are causative for CMT2/dHMN and reside in the well-conserved ACD (Nefedova et al. 2015). Inheritance is dominant in most of the patients, but de novo mutations are frequent and rare cases of recessive transmission have been observed. Why mutations in this ubiquitously expressed molecular chaperone lead to almost exclusive neuropathic phenotypes still remains obscure.

**Table 2:** Mutations reported in HSPB1, HSPB3, and HSB8 involved in hereditary neuropathies (adapted from Adriaenssens et al. 2017)

Gene	Disease	Mutation	Type of mutation	Domain	Patients, *n*^a^	Reference
HSPB1	CMT2/dHMN	P7S	Missense	N-Terminal	1	Echaniz-Laguna et al. (2017a)
		P7R	Missense	N-Terminal	2	Fortunato et al. (2017)
		G34R	Missense	N-Terminal	11	Capponi et al. (2011)
		P39L	Missense	N-Terminal	10	Houlden et al. (2008), Capponi et al. (2011), Echaniz-Laguna et al. (2017a)
		E41K	Missense	N-Terminal	2	Capponi et al. (2011)
		G53D	Missense (recessive)	N-Terminal	1	Echaniz-Laguna et al. (2017a)
		L58fs*105	Frameshift	N-Terminal	1	Echaniz-Laguna et al. (2017a)
		A61fs*100	Frameshift	N-Terminal	1	Echaniz-Laguna et al. (2017a)
		G84R	Missense	N-Terminal	3	Houlden et al. (2008), James et al. (2008)
		L99M	Missense (recessive)	Alpha-crystallin	1	Houlden et al. (2008)
		R127W	Missense	Alpha-crystallin	16	Evgrafov et al. (2004), Tang et al. (2005), Dierick et al. (2008), Echaniz-Laguna et al. (2017a)
		R127L	Missense	Alpha-crystallin	3	Ylikallio et al. (2015)
		Q128R	Missense	Alpha-crystallin	1	Echaniz-Laguna et al. (2017a)
		S135F	Missense	Alpha-crystallin	40	Evgrafov et al. (2004), Chung et al. (2008), Houlden et al. (2008), Echaniz-Laguna et al. (2017a)
		S135C	Missense	Alpha-crystallin	6	Benedetti et al. (2010), Oberstadt et al. (2016)
		S135Y	Missense	Alpha-crystallin	10	Ylikallio et al. (2014), Rossor et al. (2017)
		R136W	Missense	Alpha-crystallin	1	Evgrafov et al. (2004)
		R136L	Missense	Alpha-crystallin	10	Capponi et al. (2011), Gaeta et al. (2012), Stancanelli et al. (2015)
		R140G	Missense	Alpha-crystallin	8	Houlden et al. (2008), Rossor et al. (2017)
		K141Q	Missense	Alpha-crystallin	4	Ikeda et al. (2009), Maeda et al. (2014)
		T151I	Missense	Alpha-crystallin	6	Evgrafov et al. (2004), Dierick et al. (2008), (Echaniz-Laguna et al. 2017a)
		S158fs*200	Frameshift	Alpha-crystallin	4	Mandich et al. (2010), Capponi et al. (2011)
		T164A	Missense	Alpha-crystallin	3	Lin et al. (2011)
		M169fs*2	Frameshift	C-Terminal	1	Ylikallio et al. (2015)
		Q175*	Nonsense	C-Terminal	17	Rossor et al. (2012), Echaniz-Laguna et al. (2017a)
		T180I	Missense	C-Terminal (IXI/V motif)	5	Luigetti et al. (2010), Capponi et al. (2011), Echaniz-Laguna et al. (2017a)
		P182A	Missense	C-Terminal (IXI/V motif)	11	Rossor et al. (2017)
		P182L	Missense	C-Terminal (IXI/V motif)	2	Evgrafov et al. (2004), Dierick et al. (2008)
		P182S	Missense	C-Terminal (IXI/V motif)	1	Kijima et al. (2005)
		S187L	Missense	C-Terminal (IXI/V motif)	1	Echaniz-Laguna et al. (2017a)
		R188W	Missense	C-Terminal (IXI/V motif)	1	Capponi et al. (2011)
HSPB3	CMT2/dHMN	R7S	Missense	N-Terminal	2	Kolb et al. (2010), Lassuthova et al. (2016)
		Y118H	Missense	Alpha-crystallin	2	Nam et al. (2018)
HSPB8	CMT2/dHMN	P90L	Missense	N-Terminal	1	Echaniz-Laguna et al. (2017a)
		N138T	Missense	Alpha-crystallin	3	Echaniz-Laguna et al. (2017a)
		K141N	Missense	Alpha-crystallin	47	Irobi et al. (2004), Tang et al. (2005), Dierick et al. (2008), Echaniz-Laguna et al. (2017a)
		K141M	Missense	Alpha-crystallin	2	Echaniz-Laguna et al. (2017a)
		K141E	Missense	Alpha-crystallin	4	Irobi et al. (2004), Dierick et al. (2008)
		K141T	Missense	Alpha-crystallin	1	Nakhro et al. (2013)

^a^The number of patients (reported in literature before the date of submission, April 2019) was determined by incorporating all patients which have undergone neurological examination and for whom the mutation was confirmed by genetic testing

To elucidate the pathological mechanisms of HSPB1 mutations, d’Ydewalle et al. (2011) developed and characterized transgenic mice overexpressing human wild-type or mutant HSPB1 (S135F and P182L) using a neuron-specific Thy1.2 promotor. From the age of 6 months onwards, both mutant HSPB1 lines showed a significant reduction of the compound muscle action potential (CMAP) amplitudes and a decreased rotarod performance with progressive worsening over time. The S135F model, but not the P182L, showed sensory abnormalities as assessed by the hot plate test and sensory nerve action potentials (SNAPs). In the presence of mutant HSPB1, α-tubulin acetylation was decreased in motor neurons and severe axonal transport deficits were observed in dorsal root ganglion (DRG) sensory neuron explants. Interestingly, using lentiviral-transduced primary motor neurons, it was confirmed that these mitochondrial transport defects also occur in motor neurons. Strikingly, this same study demonstrated that only the retrograde transport of mitochondria (but not other cargo) was affected (Kalmar et al. 2017). This suggests that mitochondrial deficits may be a very specific and important factor in the pathogenesis. Administration of histone deacetylase 6 (HDAC6) inhibitors resulted in an increase of α-tubulin acetylation and restored the axonal transport defects in vivo. The restoration of the acetylated α-tubulin and accompanied axonal transport led to an improvement of the neuromuscular phenotype (d’Ydewalle et al. 2011). However, despite restoration of the axonal transport defects back to wild type, the motor performance and electrophysiological parameters of these animals were only partially restored. This implies that, besides α-tubulin acetylation and axonal transport, additional pathways might contribute to the disease. This is not surprising given the pleiotropic functions HSPB1 fulfils but it offers a window for additional therapies to improve the phenotype further. Interestingly, Regenacy Pharmaceuticals and the Charcot-Marie-Tooth Association teamed up in 2018 to further develop the therapy with ricolinostat (a selective HDAC6 inhibitor) and evaluate its potential for the clinic. So exciting times are ahead with potentially the first treatment for HSPB1 in clinical trial soon.

Some of the first observations made for HSPB1 mutants highlighted important aspects of the underlying molecular biology. Like other sHSPs, HSPB1 can form a wide range of oligomers which are composed of monomers and dimers that dynamically exchange between oligomers (Baldwin et al. 2011). However, some HSPB1 ACD mutants (e.g., R127W, S135F, and R136W) were shown to reside more in the monomeric state than the wild-type protein. Moreover, these same mutants were found to have a higher chaperone activity (Almeida-Souza et al. 2010). Conversely, disease-causing mutants that did not influence monomerization showed either no change (T151I) or a decrease (P182L/S) in activity (Almeida-Souza et al. 2010; Almeida-Souza et al. 2011; Chalova et al. 2014). Based on these results, it was proposed that the monomer could be an active form of HSPB1 while other studies suggested that its chaperone activity remains with the dimer (Jovcevski et al. 2015). It has long remained enigmatic whether the monomeric form of HSPB1 truly contributes to its chaperone function. Monomer formation requires reduction of the cysteine residue that otherwise forms an inter-dimer disulphide bond. Now, Alderson et al. (2019) show that the dissociation of HSPB1 into the monomeric state under reducing conditions is accompanied by increased chaperone activity and partial unfolding of the ACD. The β-strands that mediate dimerization become partially unfolded and highly dynamic in the monomer. Interestingly, a number of HSPB1 mutations cluster to this dynamic region of the monomer. This further supports a role for the monomer and highlights the need to generate a comprehensive understanding of its role in order to decipher the molecular basis of CMT and dHMN.

Besides the increased chaperone activity and increased monomerization, these ACD mutants also increased the affinity of HSPB1 for its clients. Whereas the interaction between wild-type HSPB1 and its clients is transient and weak, making it very difficult to capture them with co-immunoprecipitation or NMR, the introduction of these ACD mutants allowed to readily capture and identify the client proteins bound to HSPB1. In doing so, tubulin was identified as one of the main interactors of HSPB1 (Almeida-Souza et al. 2010; Almeida-Souza et al. 2011). We found that HSPB1 binds along the surface of microtubules and has an important role in maintaining them stabile yet dynamic. Furthermore, HSPB1 not only stabilizes already formed microtubules but also assists during non-centrosomal nucleation of microtubules (Almeida-Souza et al. 2013). As a consequence of the increased affinity for tubulin, the ACD mutants bind stronger to microtubules and cause an overstabilization. These molecular insights into the interaction between HSPB1 and tubulin led to the observation of altered tubulin acetylation in vivo, yielding HDAC6 inhibitors as a new candidate therapy for axonal CMT2 caused by HSPB1 mutations (d’Ydewalle et al. 2011).

In an attempt to explain why HSPB1 mutations only affect such a specific cell type, the study of Holmgren et al. (2013) seems of particular interest. Here, it was demonstrated that in lentiviral-transduced SH-SY5Y cells overexpressing wild-type or mutant HSPB1, the transport of neurofilaments is affected. These neurofilament transport defects were suggested to be caused by a decreased interaction between neurofilament and kinesin due to increased phosphorylation of neurofilaments by CDK5. Interestingly, reducing the CDK5 expression with short hairpin RNAs restored neurofilament phosphorylation and its interaction with kinesin. Similarly, using pharmacological inhibition of CDK5 with roscovitine, the axonal transport of neurofilament was restored. Given the tissue-specific expression of neurofilament and the observation that mutations in neurofilament light (NFL) also cause CMT, this inhibition of CDK5 may provide an appealing pharmacological target.

Besides its canonical chaperone function, HSPB1 is involved in numerous cellular processes, such as apoptosis (Charette et al. 2000; Qi et al. 2014), cytoskeleton dynamics (Der Perng and Quinlan 2004; Almeida-Souza et al. 2011; Clarke and Mearow 2013), translation (Cuesta et al. 2000; Geuens et al. 2017), and autophagy (Tang et al. 2011; Matsumoto et al. 2015). Haidar et al. (2019) recently showed that different mutations in HSPB1 (R127W, S135F, and P182L) lead to an impairment of autophagy, a ubiquitous multi-step process which prevents the accumulation of misfolded proteins and damaged organelles in the cytoplasm. The autophagy receptor SQSTM1/p62 (sequestosome-1 or ubiquitin-binding protein p62) was identified as an interactor of wild-type and mutant HSPB1, the latter showing an increased binding affinity for this client. SQSTM1/p62 clusters ubiquitinated proteins in the cytoplasm in small dense round formations (known as p62 bodies), which form a scaffold for the formation of a double-membraned vesicle referred to as the autophagosome (Yang and Klionsky 2010). HSPB1 mutant cells displayed an impairment of autophagosome formation due to a decrease in the formation of p62 bodies, suggesting that HSPB1 fulfils a regulatory role in autophagy through its interaction with SQSTM1/p62. The autophagy deficits were also confirmed in motor neurons differentiated from patient-derived induced pluripotent stem cells (iPSCs), thereby indicating that the impairment of autophagy might be another pathomechanism by which mutations in HSPB1 cause a peripheral neuropathy.

**Fig. 2:**
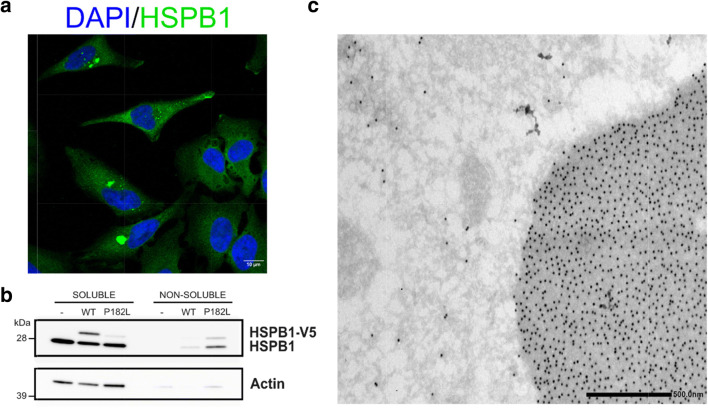
**a** Immunocytochemistry of HeLa cells which were lentiviral-transduced with the HSPB1-P182L mutant and decorated with anti-V5 antibodies to visualize HSPB1. Bright green areas are HSPB1 aggregates. **b** Western blot analysis of the same cells with separation of the NP-40 soluble and insoluble fractions, demonstrating the transition of the HSPB1-P182L from the soluble to the non-soluble fraction. **c** Immuno-electron microscopy of HeLa cells transduced with GFP-tagged HSPB1 and stained with an anti-GFP antibody. Black dots represent gold-labeled antibodies with the gray and electron-dense GFP-positive HSPB1 protein aggregate on the right, which is located in the cytosol (unpublished results)

Most of the studies mentioned above focused on ACD mutants for which it is now well established that they increase the affinity for their molecular clients and subsequently disturb the function of those clients. However, in the C-terminus of HSPB1 lies another well-studied mutation. With a disease onset in the first decade of life and a relatively rapid disease progression, the P182L mutation is one of the most severe mutations in HSPB1 (Evgrafov et al. 2004). This P182L mutant resides in the conserved IXI/V motif and increases the oligomer size drastically. In cells, this leads to the formation of very large aggregates which become insoluble (Fig. 2) (Ackerley et al. 2006; Chalova et al. 2014). As a consequence of these altered biophysical properties, it was shown that this mutant is more retained to the neuronal cell body. Also neurofilament medium and p150 were found to have an altered distribution in primary cortical neurons transduced with HSPB1-P182L (Ackerley et al. 2006). In addition, Geuens et al. (2017) reported the RNA-binding protein named poly(C)binding protein 1 (PCBP1) as a novel interaction partner for HSPB1, to which the P182L bound stronger. As a consequence of this aberrant interaction between HSPB1-P182L and PCBP1, there was a loss of translational repression by PCBP1 on its mRNA targets. Surprisingly, a number of CMT-associated genes were among its targets and were found to have an altered expression in a mutant HSPB1-P182L background. Although it is clear that the P182L mutant causes the most severe neuropathy phenotype and obtains certain unique properties due to the mutation (compared with ACD mutants), conclusive data is missing to explain this genotype-phenotype correlation. Based on all the studies mentioned above, it seems that different mutations can impact the affinity of HSPB1 towards different clients, indicating a potential difference in underlying pathomechanism.

In contrast to the ACD and C-terminus, N-terminal mutations remain under-studied. Interestingly, McDonald et al. (2012) suggested an important role for the N-terminus in binding (at least some) client proteins. Physico-chemical analysis of N-terminal HSPB1 mutants (G34R, P39L, and E41K) in vitro showed an increased stability of large HSPB1 homooligomers, reduced phosphorylation-dependent dissociation, and a decrease in chaperone capacity, thereby disturbing normal functioning of HSPB1 (Muranova et al. 2015). Interestingly, although poorly conserved, the N-terminal domain contains a core RLFDQxFG motif present in many members of the sHSP family. Replacement of the arginine (R) by alanine induced spectral changes (absorbance and fluorescence spectrum) and/or changes of temperature-induced aggregation only in the case of HSPB1 and HSPB8, but not for HSPB4, HSPB5, or HSPB6 (Shatov et al. 2018). Finally, one of the few molecular studies on the impact of N-terminal mutants in cells demonstrated that the P39L mutant had a similar effect on mitochondria as the S135F (Kalmar et al. 2017).

In summary*,* research on HSPB1 mutants has made a lot of progress over the last decade. A number of potential pathomechanisms have been unveiled, such as the impairment of autophagy, altered chaperone activity, the tendency to aggregate, and axonal transport deficits. As many of these results were only obtained *in cellulo*, it will be critical for future studies to transition these insights into the in vivo situation and to assess for each of these pathways individually whether or not they should be considered contributing factors to the disease.

### HSPB3

In 2010, the first mutation in HSPB3 (R7S) was identified in two siblings diagnosed with dHMN (Kolb et al. 2010). The same mutation was identified later in a Czech patient by a gene panel analysis (Lassuthova et al. 2016). A functional follow-up study reported that the R7S mutation had no pathogenic effect upon overexpression in avian motor neurons (La Padula et al. 2016). Only recently, a second mutation (Y118H) in HSPB3 was found in two patients presenting with CMT2 symptoms (Nam et al. 2018). Since little is known about the biological function of HSPB3, it is hard to predict the impact of these mutations. In addition, the fact that two large cohort studies on sHSPs did not yield any additional mutations (Capponi et al. 2011; Echaniz-Laguna et al. 2017a) leaves it uncertain whether those HSPB3 variants are truly pathogenic.

Like other sHSPs, HSPB3 is able to form oligomeric structures, which are uniquely composed of hetero-tetramers of HSPB2 and HSPB3 in a 3:1 ratio (Sugiyama et al. 2000; den Engelsman et al. 2009). The R7 residue is close to N-terminal IXI/V motifs that are proposed to bind the β4/β8 pocket of a HSPB2 molecule (Clark et al. 2018). It is therefore suggestible that the replacement of the positively charged arginine by a polar serine affects the ability of the protein to oligomerize and fulfil its biological function. However, data obtained from mass spectrometry and analytical ultracentrifugation of HSPB2/HSPB3-R7S indicated that the mutation has only a minor impact on the hetero-tetramer assembly and stoichiometry (Clark et al. 2018)*.* The Y118 residue lies at the border of the shared groove between HSPB2 and HSPB3, which is important for dimerization. Whether the Y118H mutation alters the HSPB2-HSPB3 interaction remains to be determined. However, another residue in this groove, R116, is mutated to proline in patients diagnosed with myopathy, which was already shown to disrupt the binding to HSPB2 when assessed by co-immunoprecipitation (Morelli et al. 2017b).

Taken together, as conclusive data is missing on the biological function of HSPB3, it is difficult to predict the consequences of mutations in the *HSPB3* gene. Moreover, the fact that only very few families have been identified with an *HSPB3* mutation, and that the pathogenicity of HSPB3 mutants is not warranted, requires the identification of additional families or patients to firmly establish its causality.

### HSPB8

Patients with HSPB8 mutations present with a very similar phenotype as patients with HSPB1-linked neuropathy, with the exception that thigh weakness, CNS involvement, and autosomal recessive transmission have not been observed. With an estimated prevalence of 5%, genetic variants in the *HSPB1* gene are a frequent cause of CMT2/dHMN, whereas *HSPB8* mutations are still rare and account for only 1% of patients. More recently, the disease spectrum caused by mutations in HSPB8 was expanded to distal myopathy (Ghaoui et al. 2016; Echaniz-Laguna et al. 2017b). Disease-causing variants in HSPB8 predominantly target the same amino acid residue (Lys141) in the highly conserved ACD. This hot-spot residue therefore seems to play an important role in the structure and function of HSPB8. In February 2019, collaborating neurologists Prof. Dr. J. Baets and colleagues performed the first autopsy of a dHMN patient with the HSPB8_K141N mutation (personal communication). The patient was a member of the original Belgian family (Timmerman et al. 1992; Irobi et al. 2004). She passed away at the age of 76 and was wheelchair bound since the age of 48. The autopsy will allow us to study in detail the neuropathological consequences of this mutation in human and assess its similarities to what has been described for the mouse.

A knock-in mouse model for the K141N missense mutation was developed, mimicking the neuropathy phenotype segregating in the originally described Belgian dHMN family (Bouhy et al. 2018; Timmerman et al. 1992). The *Hspb8*^*K141N/K141N*^ mice showed a progressive and significant reduction of the compound muscle action potential (CMAP) amplitudes from the age of 6 months onwards, which was further supported by a significant decrease in performance on the rotarod. At the molecular level, Hspb8 aggregates were detected by immunohistochemistry in sciatic nerves of 15 months old *Hspb8*^*K141N/K141N*^ mice but not in *Hspb8*^*+/+*^ animals. Additionally, in the muscle of 12-month-old *Hspb8*^*K141N/K141N*^ mice, we also detected Hspb8-positive aggregates associated with desmin and αβ-crystallin. Interestingly, the *Hspb8*^*−/−*^ mice did not develop any motor deficit or electrophysiological abnormalities. Aged *Hspb8*^*−/−*^ mice showed only minor structural irregularities in the muscle which remained subclinical as the mice performed as good as age-matched controls on the rotarod.

The absence of any neuropathological phenotype in the *Hspb8*^*−/−*^ mice might be explained by (i) the absence of Hspb8 aggregates, (ii) activation of compensatory mechanisms mimicking the role of Hspb8, and (iii) the absence of any additional toxic gain-of-function consequences of the K141N mutant. We have previously shown that mutant human HSPB8 is prone to aggregation in patient fibroblasts (Irobi et al. 2012). However, there is so far no clear consensus on how these aggregates contribute to the neurodegeneration (Adriaenssens et al. 2017; Carra et al. 2019). Regarding the molecular compensation, it is a principle that is frequently observed within the class of the sHSPs (Carra et al. 2019). For instance, multiple sHSPs were shown to bind BAG3 (BCL2-associated athanogene 3; the main binding partner of HSPB8), of which HSPB8 has the highest affinity (Fuchs et al. 2009; Hishiya et al. 2011; Morelli et al. 2017a). It is therefore likely that in the absence of HSPB8, other sHSPs are able to replace HSPB8 and interact with BAG3 to maintain cellular proteostasis. Interestingly, Lys141 mutations were shown to both increase (Echaniz-Laguna et al. 2017a) and decrease (Carra et al. 2010; Shemetov and Gusev 2011) the binding with BAG3. It remains unclear why these studies led to different results, but it is clear that the HSPB8-BAG3 interaction is directly affected by mutations at this lysine residue. Intriguingly, dominant mutations in the HSPB8-interacting IPV motif of BAG3 are also known to cause myofibrillar myopathy and axonal neuropathy (Selcen et al. 2009; Odgerel et al. 2010; Shy et al. 2018), further implying an important role for this protein complex in neuromuscular homeostasis.

HSPB8 acts as a chaperone in the removal of aggregates through chaperone-assisted selective autophagy (CASA) together with the co-chaperone BAG3, HSP70/HSC70 (heat shock protein/cognate 70), and SQSTM1/p62 (Carra et al. 2008). Client proteins are polyubiquitinated by the E3 ligase STUB1/CHIP (STIP1 homology and U-box containing protein 1) and subsequently sequestered into cytoplasmic puncta that are labeled with SQSTM1/p62 (also called p62 bodies). SQSTM1/p62 functions as a multi-adaptor protein by binding simultaneously to ubiquitin and the autophagosome-associated protein MAP1LC3/LC3 (microtubule associated protein 1 light chain 3), allowing misfolded proteins to be inserted into autophagosomes, followed by lysosomal degradation (Ciuffa et al. 2015; Wurzer et al. 2015; Cha-Molstad et al. 2017; Zaffagnini et al. 2018; Turco et al. 2019). Overexpression of wild-type HSPB8 in neuronal cells led to an increased colocalization of autophagosomes with lysosomes while overexpression of mutant HSPB8 (K141N) reduced colocalization between autophagosomes and lysosomes (Kwok et al. 2011). In cells from two dHMN patients with the K141E mutation, a similar impairment of autophagy was observed. Guilbert et al. (2018) reported a role for HSPB8 in ubiquitinated microaggregate formation. Depletion of HSPB8 impaired the formation of these microaggregates and the early formation of p62 bodies. Although they did not include mutant HSPB8 in their experiments, a similar mechanism might explain the autophagic deficits observed in the *Hspb8*^*K141N/K141N*^ mouse model (Bouhy et al. 2018). Interestingly, this is the first time that the two sHSPs, HSPB1 and HSPB8, evidently share the same pathomechanism as their activities merge in SQSTM1/p62 modulation.

The HSPB8-BAG3-HSP70 chaperone complex also helps to maintain the integrity and dynamics of stress granules (Ganassi et al. 2016), which are membrane-less ribonucleoprotein assemblies that form upon proteotoxic stress (Kedersha and Anderson 2002). In contrast to HSPB8, which is immediately recruited into forming stress granules, HSPB1 does not seem to be crucial for the formation of stress granules because neither short hairpin RNA-mediated knockdown nor CRISPR knock-out of HSPB1 affected the formation of stress granules (unpublished data). However, HSPB1 is specifically recruited to aberrant stress granules that contain misfolded proteins, suggesting that it is an important player in their disassembly and thereby protects the cell from toxicity (Mateju et al. 2017). So far, little is known about the influence of neuropathy-linked mutations in HSPB1 and HSPB8 on the formation and dynamism of stress granules. While it is well established that stress granule dysregulation contributes to the motor neuron disease amyotrophic lateral sclerosis (ALS) (Marrone et al. 2018), it will be an interesting topic to explore further in the context of CMT2/dHMN.

Unrelated to the classical chaperone model, the HSPB8-BAG3 complex was reported to be involved in the phosphorylation of eIF2α (eukaryotic initiation factor 2 alpha) (Carra 2009; Carra et al. 2009) and the remodeling of actin-based mitotic structures (Fuchs et al. 2015). Due to the broad range of functions HSPB8 exerts, it is difficult to pinpoint which functional impairment caused by mutant HSPB8 is responsible for the neuropathic and myopathic disorders.

Until now, most (if not all) studies that attempted to unravel the pathomechanism for HSPB8 were focused on mutants targeting the Lys141 residue. However, missense mutations in *HSPB8* not targeting this Lys141 hot-spot were also reported (Echaniz-Laguna et al. 2017a). Both mutations (P90L and N138T) give rise to an identical motor-predominant phenotype. Surprisingly, none of these mutations changed the affinity of HSPB8 for BAG3. This might suggest that either (i) the protein complex is still intact but that HSPB8 may have become inactive, (ii) the underlying pathomechanism might differ, or (iii) the interaction with BAG3 is not relevant in disease context, although the latter idea seems less appealing at the moment as mutations in BAG3 may also cause CMT2/dHMN (Shy et al. 2018). Moreover, in a recent attempt to identify new interaction partners through immunoprecipitation-mass spectrometry (IP-MS), no additional binding partners, apart from BAG3, were found for wild-type or mutant HSPB8 (unpublished results). So further follow-up is required to unambiguously demonstrate the pathogenicity of these non-hot-spot mutations.

In summary, it remains striking how many of the HSPB8 mutations target the same amino acid. Yet, the molecular consequences of these mutations are still poorly understood. For instance, it has been suggested that these mutants lead to protein aggregation of HSPB8. However, it is unknown whether this is driven by HSPB8 itself due to its altered biophysical properties or whether HSPB8 is subjected to an aggregation-stimulating environment such as stress granules (a membrane-less compartment with a high abundance of hydrophobically exposed residues). These outstanding questions, along with the discovery of yet unknown roles for HSPB8, are topics that will hopefully be enlightened over the following years. In addition, the availability of a mouse model that mimics the patient’s phenotype offers the potential to verify in vitro obtained hypotheses and the potential to evaluate candidate therapies for HSPB8 patients.

## Small heat shock proteins as modifiers of aggregation-prone neurodegeneration

Many neurodegenerative disorders are characterized by the accumulation of protein deposits. Such protein deposits are generated through the assembly of insoluble fibrils from otherwise soluble proteins. These fibrils can be formed by different proteins, yet they adopt a similar cross-β sheet conformation (Eisenberg and Jucker 2012). This process is referred to as amyloidosis and these amyloid structures are among the most stable protein conformations known to date. In some cases, they can even compete with steel in terms of strength (Smith et al. 2006). Amyloid fibrils also occur naturally, and thus not only in disease, as they are known to fulfil biological functions such as pigment formation, neuronal signaling, and hormone release (Fowler et al. 2007; Maji et al. 2009; Roan et al. 2011; Roan et al. 2017; Rice et al. 2019).

Cells thus contain a number of metastable proteins that are more vulnerable for aggregation. In order to prevent these proteins from aggregating, they are subject to tight regulation. However, if this process becomes dysregulated, it may cause fatal neurodegenerative diseases such as Alzheimer’s, Parkinson’s, and Huntington’s disease (Blancas-Mejia and Ramirez-Alvarado 2013; Chuang et al. 2018). Thus, amyloidogenesis does not necessarily require mutations in these metastable proteins to occur. However, mutations in such genes frequently increase their tendency to fibrillize and aggregate. Worryingly, once formed, these amyloid fibrils can self-replicate and transfer between cells; thereby spreading across tissues (Guo and Lee 2014; Goedert et al. 2017).

To control these intrinsic protein instabilities, nature has developed mechanisms to counter uncontrolled protein aggregation (Muchowski and Wacker 2005; Lindberg et al. 2015). However, under certain stressful conditions, these protective systems can be overloaded or become compromised leading to the formation of amyloid-like structures. Below we will discuss some of the most frequent neurodegenerative disorders that are characterized by protein aggregation and highlight the role and potential of sHSPs in controlling the aggregation process.

### Alzheimer’s disease

Alzheimer’s disease is the most common cause of dementia worldwide and is typically recognized by progressive memory loss and confusion. Pathologically, Alzheimer’s disease is characterized by extracellular amyloid plaques (amyloid-beta/Aβ) and intracellular neurofibrillary tangles (hyperphosphorylated tau) (Selkoe 2001). It is still under debate whether only extracellular Aβ accumulation contributes to the disease or whether also intracellular Aβ pools do (Benilova et al. 2012; Selkoe and Hardy 2016). One view is that amyloid fibrils or their soluble misfolded oligomeric antecedents are directly toxic to cells. This gain-of-function mechanism is supported by the observation that synthetically engineered amyloids or soluble misfolded oligomers with no native function are directly toxic to cells (Bucciantini et al. 2002; Olzscha et al. 2011). However, this does not exclude the possibility that loss-of-function may also contribute to the disease progression as the co-aggregation of other proteins may reduce their availability in the cytosol. Both mechanisms are not necessarily mutually exclusive and may thus contribute to the pathogenesis.

#### Alzheimer’s disease—amyloid-beta/Aβ

The amyloid precursor protein (APP) is a transmembrane protein that is processed by different secretases. However, due to improper cleavage by β- and γ-secretases, neurotoxic Aβ peptides may be formed. These peptides go on to form insoluble amyloid fibrils which accumulate in extracellular senile plaques, a hallmark of Alzheimer’s disease.

In Alzheimer patients, both HSPB5 and HSPB8 are upregulated in the brain and found to colocalize with Aβ in senile plaques (Shinohara et al. 1993; Renkawek et al. 1994; Wilhelmus et al. 2006b). HSPB5 was also shown to colocalize with Aβ in the eyes of patients, being the tissue with the highest HSPB5 expression (Goldstein et al. 2003). Similarly, HSPB1 was found to localize to plaques in transgenic mouse models of Alzheimer’s disease (Ojha et al. 2011b).

The colocalization of sHSPs with senile plaques could be explained by two mechanisms. Either they fulfil a protective role in these plaques or they were trapped and co-aggregate along with other proteins. To distinguish between these two possibilities (protective versus co-aggregating), cellular and mouse models were employed to study this process in more detail. For instance, neurons cultured from HSPB1-deficient mice were shown to be more sensitive to Aβ oligomer-mediated toxicity (Ojha et al. 2011b). Similarly, crossing HSPB2/HSPB5 double deficient mice with a transgenic Alzheimer’s disease model worsened the phenotype (Ojha et al. 2011a). So, the lack of sHSPs seems to aggravate the Alzheimer’s symptoms. In line with this, a transgenic Alzheimer’s mouse model subjected to physical activity showed increased expression of HSPB5 accompanying the hippocampal improvements (Huttenrauch et al. 2016). Crossing the APPswe/PS1dE9 mouse model for Alzheimer’s disease with a mouse model that overexpresses HSPB1 led to significant improvements in spatial learning and electrophysiological parameters. Moreover, these mice had fewer amyloid plaques in the brain (Toth et al. 2013). These in vivo studies suggest that the lack of sHSPs aggravates the phenotype, whereas an increased expression alleviates the symptoms. This indicates that sHSPs may provide protection.

To understand the underlying principles of this potential protective role, a number of in vitro studies have probed the interaction between sHSPs and Aβ. For instance, HSPB1, HSPB5, HSPB6, and HSPB8 were found to inhibit oligomerization of Aβ monomers and prevent oligomers to grow further into fibrils (Wilhelmus et al. 2006a; Wilhelmus et al. 2006b; Dehle et al. 2010; Narayan et al. 2012). Likewise, HSPB4 (α-crystallin) that is naturally only expressed in the eye lens was also shown to inhibit fibril formation by Aβ and to counter the Aβ-associated cytotoxicity (Santhoshkumar and Sharma 2004). Mechanistically, HSPB5 was suggested to compete with Aβ monomer-monomer interactions as shown by solution-state NMR spectroscopy (Narayanan et al. 2006). For HSPB6, it was suggested that it provides neuroprotection by binding adjacent to the oligomerization domain of Aβ, preventing its aggregation (Cameron et al. 2014). However, besides countering the initial seeding and formation of Aβ oligomers, sHSPs were also shown to suppress the elongation phase of already formed amyloid fibrils. This was for instance elegantly demonstrated in a study employing immuno-electron microscopy, which demonstrated that HSPB5 can bind along the entire length of amyloid fibrils (Shammas et al. 2011). This mode-of-action is interesting as it resembles how HSPB1 was shown to bind and stabilize microtubules (Almeida-Souza et al. 2011; Almeida-Souza et al. 2013). In the case of microtubules, this lateral binding was shown to be both sufficient and necessary to maintain the intimate balance between microtubule stability and dynamics. However, in the case of amyloid fibrils, it remains to be studied further how this lateral binding can prevent fibril growth.

#### Alzheimer’s disease—tau

Another hallmark of Alzheimer’s disease, besides the extracellular senile Aβ-plaques, is the formation of intracellular neurofibrillary tangles. These intracellular protein deposits are composed of hyperphosphorylated tau (Caughey and Lansbury 2003). As with many forms of amyloidosis, it is still under debate whether the end stage, neurofibrillary tangles in this case, causes neurodegeneration or whether toxicity is also induced by smaller, soluble tau species, such as tau oligomers (Wang and Mandelkow 2016).

Tau is encoded by the microtubule-associated protein tau (MAPT) gene and causes neurodegenerative diseases referred to as tauopathies (Lee et al. 2001) including some forms of Alzheimer (Ballatore et al. 2007). Tau oligomers have been detected in brain tissues from Alzheimer’s patients and their level shows a relation with the degree of memory deficits (Berger et al. 2007).

Both HSPB1 and HSPB5 were shown to colocalize with tau fibrils in tauopathy brains and the amount of HSPB1 and HSPB5 was shown to correlate inversely with the levels of granular tau (Nemes et al. 2004; Sahara et al. 2007; Bjorkdahl et al. 2008). In mice, overexpression of HSPB1 was found to be partially protective against tauopathy (Shimura et al. 2004).

To elucidate the underlying principles, mechanistic in vitro studies demonstrated that HSPB1 can delay tau fibril formation by weakly interacting with early aggregating species. However, this binding is not enhanced under aggregation-promoting conditions suggesting that HSPB1 can delay but not prevent fibril formation (Baughman et al. 2018). Interestingly, a physical interaction between HSPB1 and tau was confirmed by co-immunoprecipitation and shown to be increased when tau was hyperphosphorylated (Abisambra et al. 2010). This increased affinity of HSPB1 for hyperphosphorylated tau may actually depend on a small subset of phosphogroups as only certain phosphomimics of tau were found to be slowed down in their aggregation by HSPB1, HSPB5, and HSPB8 (Mok et al. 2018). At the atomic level, very similar models were proposed for HSPB1 and HSPB5 in which competition occurs between the IPV motif of a sHSP and tau for the β4-β8 binding site of HSPB1 or HSPB5 (Freilich et al. 2018; Liu et al. 2018). It was noted that increasing concentrations of tau can displace the C-terminal IPV motif and thereby take over the β4-β8 binding site in the ACD of HSPB1. However, the ACD binding alone is not sufficient to prevent tau aggregation. The additional contacts with the N-terminus are required, which suggests that the ACD fulfils a role as client sensor while the N-terminus is required for the chaperone function (Freilich et al. 2018).

### Parkinson’s disease—alpha-synuclein/α-synuclein

Parkinson’s disease is the second most common neurodegenerative disorder after Alzheimer’s disease. Clinical diagnosis is often difficult considering the large number of motor and non-motor symptoms patients can present. Parkinson’s disease is characterized by neuronal loss in specific areas of the *substantia nigra* and the widespread aggregation of protein. A major constituent of these intracellular aggregates is α-synuclein. Alpha-synuclein is a small (14 kDa) intrinsically disordered protein encoded by the *SCNA* gene. Neither the loss of dopaminergic neurons in the *substantia nigra* nor the deposition of α-synuclein is specific for Parkinson’s disease; however, the combination of both is sufficient to make a definitive diagnosis (Poewe et al. 2017).

There is compelling evidence that α-synuclein contributes to both sporadic and familial cases of Parkinson’s disease. On the one hand, mutations in *SCNA* cause early-onset Parkinson’s disease due to the production of highly aggregation-prone variants of α-synuclein (Trinh and Farrer 2013). On the other hand, mutations in a non-coding distal enhancer element of *SCNA* result in an increased expression of α-synuclein and are considered risk factor for Parkinson’s disease (Soldner et al. 2016). So due to genetic mutations, increased abundance, or still unknown factors, α-synuclein may transition from soluble monomers to oligomers which further develop into insoluble fibrils.

Multiple sHSPs have been reported to be present in protein aggregates mainly composed of α-synuclein, which are also referred to as Lewy bodies (Lowe et al. 1990; Iwaki et al. 1992; Mizutani et al. 1998; McLean et al. 2002; Outeiro et al. 2006; Waudby et al. 2010; Bruinsma et al. 2011; Shammas et al. 2011). sHSPs were shown to counter α-synuclein aggregation in vitro and, so far only tested for HSPB5, in vivo (Bruinsma et al. 2011; Duennwald et al. 2012; Prabhu et al. 2012; Tue et al. 2012; Alderson et al. 2019). Moreover, the overexpression of HSPB1 and HSPB5 reduces intracellular aggregation and counters cytotoxicity induced by α-synuclein (McLean et al. 2002; Klucken et al. 2004; Zourlidou et al. 2004; Outeiro et al. 2006; Cox and Ecroyd 2017).

The ability of HSPB1 and HSPB5 to inhibit fibril formation was found to be dependent on the rate of aggregation. Faster aggregation (shorter lag phase) led to reduced inhibition by the sHSPs (Cox et al. 2016). Similar to the binding of HSPB5 to Aβ fibrils, HSPB1 was also found to bind along the surface of α-synuclein fibrils and thereby decreasing their hydrophobicity (Cox et al. 2018). Interestingly, the HSPB5 interaction domain was mapped to the N-terminus of α-synuclein, which is involved in membrane binding (Fusco et al. 2014; Liu et al. 2018). So besides countering α-synuclein aggregation, sHSPs may also modulate the way α-synuclein induces neurotoxicity.

### PolyQ repeat expansion–associated neurodegenerative diseases

A large group of neurodegenerative diseases is characterized by a DNA repeat expansion. These expanded repeats originate at the genomic level where specific nucleotide repeats challenge the DNA replication machinery which can lead to slippery events and expansion of the repeat sequence. This can occur in both coding and non-coding regions. Surprisingly, both can give rise to protein products which subsequently contain repeats of specific amino acids. The most common repeat expansions code for stretches of glutamine and are therefore referred to as polyQ. The best known example of a non-coding repeat expansion leading to a neurodegenerative disease is found in the *C9orf72* gene (this will be discussed under “ALS—C9orf72”). The expansion of repeat regions in the genome may therefore produce protein products which were not necessarily aggregation-prone initially; however, due to the introduction of a long repeat of specific amino acids, it may have acquired novel properties that make them aggregation-prone. Not surprisingly, these peptide repeats can form a significant challenge for chaperone systems as these protein products do not occur naturally. Below we will discuss different polyQ diseases, linked to different neurodegenerative disorders, and highlight where and how sHSPs might be involved in their surveillance.

#### PolyQ repeat expansion—spinocerebellar ataxia

Spinocerebellar ataxias are a large group of neurodegenerative disorders of which the most prevalent ones are caused by the expansion of a glutamine-encoding CAG repeat in the respective genes (Paulson et al. 2017). Their inheritance pattern is mostly autosomal dominant and the repeat size is linked to the disease penetrance (Ashizawa et al. 2018). Patients typically present with gait imbalance associated with limb incoordination and problems with gross and fine motor skills.

Through a genome-wide screen in *Drosophila* for modifiers of Ataxin-3, HSPB5 was identified as modifier for SCA3(78)Q-induced neurodegeneration (Bilen and Bonini 2007). Similarly, the overexpression of Dm-HSP67Bc (the HSPB8 ortholog in *Drosophila*) and human HSPB8 was found to counter SCA3(78)Q-mediated toxicity in the fly eye while downregulation of Dm-HSP67Bc significantly worsened the phenotype (Carra et al. 2010). In vitro, side-by-side comparison of all sHSPs demonstrated that besides HSPB8, also HSPB7 and HSPB9 are potent chaperones countering polyQ-induced aggregation of Ataxin-3 (Vos et al. 2010).

Mechanistically, HSPB5 was shown to bind to the Josephin domain of Ataxin-3, thereby inhibiting initial aggregation (Masino et al. 2004; Robertson et al. 2010). However, HSPB5 was found inactive against already formed insoluble fibrils of Ataxin-3. In addition to directly binding and modulating the aggregation process, HSPB1 was also shown to counter polyQ-induced cell death by responding to the ROS production (Wyttenbach et al. 2002).

#### PolyQ repeat expansion—Huntington

Huntington’s disease is an autosomal dominant neurodegenerative disorder characterized by symptoms as chorea and dystonia, the lack of coordination, cognitive decline, and behavioral difficulties. The disease results from a polyQ repeat expansion in the N-terminus of huntingtin. In healthy individuals, this CAG repeat does not exceed 35 copies. However, in individuals with 41 or more copies, it gives rise to the full penetrant neurodegenerative disease (The Huntington’s disease collaborative research group, 1993).

Using mutant huntingtin exon-1 as a proxy, it was found that HSPB7 is the most potent sHSP to suppress polyQ aggregation in vitro (Vos et al. 2010). Mechanistically, it was shown that HSPB7 suppresses the formation of new aggregates rather than mediating their degradation (Eenjes et al. 2016). Similarly, HSPB8, its *Drosophila* ortholog Dm-HSP67Bc, and the human HSPB8-BAG3 protein complex were all shown to have chaperone activity towards polyQ repeats in huntingtin and are capable of reducing the amount of SDS-insoluble huntingtin (Carra et al. 2005; Carra et al. 2009; Carra et al. 2010). Interestingly, while HSPB6, HSPB8, and HSPB9 were most potent against short (43Q) polyQ stretches of huntingtin, HSPB7 was more potent against the longest (119Q) polyQ stretches (Vos et al. 2010). Similar observations were made for mutant androgen receptor (ARpolyQ; causative for spinal and bulbar muscular atrophy) where HSPB8 was effective against short polyQ repeats (AR.Q46) but much less against longer AR.Q112 repeats (Carra et al. 2013). In contrast, HSPB1 and HSPB5 seemed to have little effect on polyQ huntingtin aggregation in vitro.

Expression of mutant huntingtin fragments in the lens of mice lacking HSPB5 accelerated the onset and severity of mutant huntingtin aggregation while overexpression of HSPB5 in a transgenic *Drosophila* model of Huntingon’s disease suppressed mutant huntingtin toxicity (Muchowski et al. 2008; Tue et al. 2012). This suggests, in contrast to the in vitro data, that HSPB5 is actually capable of providing protection against Huntington’s polyQ aggregation.

Interestingly, mutant huntingtin inhibits HSPB5 expression in astrocytes of the HD140Q mouse model due to a decreased activity of the SP1 transcription factor (Hong et al. 2017). This decrease in HSPB5 was suggested to lead to a reduction of exosome (extra-cellular vesicles) release, a factor that is considered to contribute to the disease pathogenesis. Restoring the HSPB5 expression either through overexpression of SP1 or HSPB5 itself was sufficient to rescue the defective exosome release from astrocytes and showed a clear reduction in mutant huntingtin aggregates (Hong et al. 2017). In line with this, in the exon-1 fragment mouse model R6/2 for Huntington’s disease, a downregulation of chaperones including HSPB5 was observed (Zabel et al. 2002; Hay et al. 2004), which was also confirmed in a different mouse model for HTT (BACHD) (Oliveira et al. 2016; Wood et al. 2019). Furthermore, using this same BACHD mouse model for Huntington’s disease, overexpression of HSPB5 in astrocytes improved motor performance and cognitive function (Gray et al. 2008; Liu et al. 2015; Oliveira et al. 2016). These improvements in behavioral deficits correlated with mitigation of neuropathological features such as decreased levels of mutant huntingtin and smaller inclusions in BACHD brains. So, overexpression of HSPB5 in astrocytes provides a non-cell autonomous pathway that modulates mutant huntingtin aggregation.

Astrocyte-derived exosomes also contain HSPB1; however, its role in relation to Huntington’s disease has provided confusing results (Nafar et al. 2016). On the one hand, a cross of the R6/2 mouse with an HSPB1 overexpression model did not lead to improvement of the R6/2 phenotype (Zourlidou et al. 2007). In contrast, other in vitro and in vivo studies actually did suggest that HSPB1 can be a protective factor for Huntington-associated polyQ repeats (Wyttenbach et al. 2002; Carra et al. 2005; Liao et al. 2008; Chen et al. 2012).

### Amyotrophic lateral sclerosis

Amyotrophic lateral sclerosis (ALS) or Lou Gehrig’s disease is a fatal neurodegenerative motor neuron disease. It affects neurons in the brain and spinal cord, leading to muscle weakness, loss of motor functions, paralysis, and breathing problems, eventually leading to death. At the molecular level, ALS is characterized by protein aggregates that accumulate as ubiquitin-positive inclusions in the cytoplasm of degenerating neurons and glia (Hardiman et al. 2017). The far majority of inclusions contain TDP-43 as their main constituent (except for patients carrying mutations in *SOD1* or *FUS)* (van Es et al. 2017). TDP-43 resides primarily in the nucleus where it fulfils a role in transcription. However, when misfolded, it aggregates in the cytosol. These TDP-43 aggregates may be toxic by themselves but may also sequester other essential cellular components. The same might be true for SOD1 or FUS aggregates. Mutations in these three genes cause familial ALS; however, FUS and TDP-43 are of particular interest as they also accumulate in sporadic ALS cases. So their presence might form an important pillar in the pathogenesis; however, the biological processes controlling their formation and/or degradation are still incompletely understood.

#### ALS—TDP-43 (TARDBP)

TAR-DNA-binding protein 43 (TDP-43) is a 414–amino acid ribonucleoprotein that resides in the nucleus but shuttles back and forth to the cytoplasm. It is both a DNA- and RNA-binding protein. It regulates transcription either through direct binding to the DNA or by interacting with components of the transcription machinery. In addition, it plays a role in splicing, RNA shuttling, and translation.

Interestingly, the vast majority of all ALS-associated mutations lay in the C-terminal low-complexity prion-like domain (PrLD) (Guo and Shorter 2017). This domain has been suggested to be important for the phase-separating nature of TDP-43; however, it may also form a potential driving site for misfolding and self-templating fibril formation. In the test tube, TDP-43 is very unstable and assembles easily into pore-shaped oligomers and fibrils. These pore-shaped oligomers share similarities with the toxic oligomers formed in other neurodegenerative diseases like α-synuclein and Aβ. Addition of these purified oligomers has been shown to cause neurotoxicity both in vitro and in vivo (Lee et al. 2011; Guo and Shorter 2017).

Although TDP-43 is very unstable in the test tube, cells seem to contain protective factors that control this aggregation-prone behavior. One such family of protective factors could be the sHSPs. For example, using a *Drosophila* model for TDP-43 neurodegeneration, it was shown that overexpression of HSP67Bc (the *Drosophila* ortholog of HSPB8) rescued the eye degeneration whereas downregulation of HSP67Bc worsened the eye phenotype (Crippa et al. 2016a). TDP-35 and TDP-25 are C-terminal fragments obtained after caspase cleavage of full-length TDP-43. They are frequently used as proxies for rapid induction of protein aggregation. In a *Drosophila* model overexpressing TDP-35, HSP67Bc rescued pupae lethality (Crippa et al. 2016a). Similarly, overexpression of human HSPB8 in neuronal cell models for ALS also protected against aggregation seeding by TDP-25, TDP-35, and TDP-43 (Crippa et al. 2010; Crippa et al. 2016a; Crippa et al. 2016b; Cicardi et al. 2018). Moreover, using an elegant approach, FDA/EMA-approved small molecules that upregulate HSPB8 were identified. Their administration to cellular ALS models greatly reduced the accumulation of misfolded TDP-43 species (Crippa et al. 2016b).

Mechanistically, HSPB8 is thought to team up with other members of the CASA complex to promote autophagosomal degradation of client proteins including TDP-43 species. Surprisingly, inhibition of dynein and thereby the autophagosomal degradation of aggregating substrates by the CASA complex were found to accelerate, instead of halt, the degradation of these client proteins due to a switch to proteasomal degradation (Teuchert et al. 2006; Zhang et al. 2007; El-Kadi et al. 2010; Cristofani et al. 2017).

The potency of other sHSPs for mutant TDP-43 clearance was tested and found, surprisingly, to be very low. So although some of them are very potent towards polyQ substrates, these same chaperones seem not potent against TDP-43 (Carra et al. 2013). This suggests that sHSPs may all have their own specific clients for which they have evolutionary developed chaperone activity.

#### ALS—C9ORF72

The most common genetic cause of familial ALS is a massive expansion of the GGGGCC hexanucleotide repeat region in the first intron of *C9orf72* (DeJesus-Hernandez et al. 2011; Renton et al. 2011; Gijselinck et al. 2012). This repeat tract is typically between 2 and 30 repeats in healthy individuals, but is increased to hundreds or even thousands in patients (Taylor et al. 2016). Intriguingly, even though this repeat is located in a non-coding region, it is still translated. This non-AUG translation, also called repeat-associated non-AUG (RAN) translation, occurs in both sense (GGGGCC) and antisense (CCCCGG) direction producing five different polymers of the predicted dipeptides, including poly glycine-alanine (poly-GA; GGGGCC), poly glycine-proline (poly-GP; GGGCCG/GGCCCC), poly glycine-arginine (poly-GR; GGCCGG), poly proline-arginine (poly-PR; CCCCGG), and poly proline-alanine (poly-PA; CCGGCC). The translation of these dipeptide repeats is likely to give rise to metastable structures as they have been shown to form inclusions, both in the cytoplasm and nucleus of neurons from patients (Ash et al. 2013; Mori et al. 2013). Interestingly, these inclusions are negative for TDP-43, even if the patients display a typical TDP-43 pathology (Al-Sarraj et al. 2011; Boxer et al. 2011; Freibaum and Taylor 2017; Vatsavayai et al. 2019).

Given that these dipeptide repeats are not naturally expressed, they may pose a particular challenge to existing chaperones. Which chaperone systems are dealing with these dipeptide repeats has not yet been fully elucidated; however, in vitro results show that overexpression of HSPB8 significantly decreases the accumulation of most dipeptide repeat insoluble species (Cristofani et al. 2018). So the family of sHSPs are putative candidates to deal with these unnatural dipeptides.

#### ALS—SOD1

The superoxide dismutase 1 (SOD1) has been linked to both familial and sporadic ALS (Deng et al. 1993; Rosen et al. 1993). For the familial cases, it is thought that SOD1 mutations cause a toxic gain-of-function leading to the disease. A hallmark of these clinical cases is the SOD1-positive inclusions composed of SOD1 protein aggregates.

Both HSPB1 and HSPB8 levels are upregulated in the muscle and/or surviving motor neurons in the spinal cord of transgenic G93A-SOD1 mice, as well as in the spinal cord of ALS patients (Vleminckx et al. 2002; Maatkamp et al. 2004; Krishnan et al. 2008; Anagnostou et al. 2010; Crippa et al. 2010; Crippa et al. 2013).

Molecularly, overexpression of HSPB1, HSPB5, and HSPB8 has been shown to reduce aggregation of SOD1 in vitro (Patel et al. 2005; An et al. 2009; Crippa et al. 2010; Yerbury et al. 2013; Capponi et al. 2016). Interestingly, CMT2/dHMN-associated mutants K141N/E were found to have decreased chaperone activity towards SOD1(G93A). Similarly, overexpression of HSPB1 wild type in astrocytes co-cultured with SOD1(G93A) motor neurons was sufficient to attenuate astrocyte-mediated cytotoxicity. However, CMT2/dHMN-associated mutants of HSPB1 (G84R and R136W) failed to provide this non-cell-autonomous protective function (Heilman et al. 2017). It requires pointing out though that the R136W mutant was shown to have an overall increased chaperone activity (Almeida-Souza et al. 2010; Almeida-Souza et al. 2011). So loss of this non-cell autonomous protection is unlikely to stem from chaperone failure. Instead, dysregulation of certain pathways due to a toxic gain-of-function may provide an explanation.

In vivo, the results are more complicated. Two studies that used a similar setup obtained contradicting results. On the one hand, the crossing of overexpressing HSPB1 mice with SOD1(G93A) mice did not yield neuroprotection, while another lab found that HSPB1 overexpression delays disease progression and increases motor unit survival (Krishnan et al. 2008; Sharp et al. 2008). Additional work is needed to decipher the origin of discrepancy between these two studies.

#### ALS—FUS

Mutations in the RNA-binding protein “fused in sarcoma” (FUS) also cause protein aggregation (Neumann et al. 2009; Deng et al. 2010). FUS shows similarities to TDP-43 as it shuttles between the nucleus and the cytosol. In addition, it can also bind both DNA and RNA (Deng et al. 2014). FUS undergoes physiological phase separation to form ribonucleoprotein (RNP) granules in the cytoplasm (Boeynaems et al. 2018). However, when FUS is mutated this process can go awry and lead to the formation of insoluble protein aggregates in the cytosol, a hallmark of the disease. Despite the similarities with other ALS-associated genes, such as TDP-43, the link between sHSPs and FUS has not yet been explored. However, given the impact of sHSPs on TDP-43 protein aggregates, it could well be that sHSPs provide protection against FUS aggregation. It will therefore be interesting to see this avenue being explored further.

## Conclusions and future perspectives

In this paper, we summarized the many links between sHSPs and neurodegenerative disorders. For HSPB1 and HSPB8, it is now well substantiated that mutations in both genes cause CMT2 and dHMN. Studying the molecular consequences of these mutations has led to the identification of multiple candidate pathways that could contribute to the disease. However, one important step that should be taken in the next years is the transition to in vivo models. A number of dysfunctional pathways that have been identified in cellular models have not yet been tested in vivo. Given the increasing number of dysfunctional pathways, which is linked to the pleiotropic functions of sHSPs, it has become unclear which of these pathways truly contribute to the pathogenesis. In vivo validation of these candidate pathways will in turn yield novel drug targets which can be tested in these same models. Moreover, the advent of patient-derived induced pluripotent stem cells is likely to become an additional player in the drug development paradigm for CMT2/dHMN (Juneja et al. 2019).

For the neurodegenerative diseases characterized by protein aggregation, the role of sHSPs is only partially understood. A number of sHSPs have been linked to various neurodegenerative disorders; however, the data is not always straightforward to interpret (as they are often obtained from different cellular and animal models). So side-by-side comparisons would aid further in assessing the potency of each of the chaperones individually, although it is to be noted here that the results may also depend on the cell type used to perform these studies. Results should therefore be interpreted with caution when obtained from in vitro models. In vivo models can provide additional information as they not only allow to assess whether a sHSP can reduce the protein aggregation but also allow to assess whether this leads to phenotypic amelioration. Finally, once it is understood which sHSPs are most potent towards which clients, it will become key to identify ways to introduce or upregulate these specific sHSPs in the desired tissues in order to obtain the desired protective effects.

Taken together, although years of research has yielded us much information about the role and link of sHSPs to neurodegeneration, the next years should allow the field to transition more towards in vivo studies and identify potential novel treatment strategies for these neurodegenerative diseases.
